# 

**Published:** 1890-10

**Authors:** 


					﻿OCTOBER. 1S©O-
OLD SERIES
Vol. xxv
& 1855 6
r K
MEW SERIES
Vol- VII —No.'s.
MM
EDIGBwUM
4OT
tl. V. M. MILLER. M.D., LL.D.
VIRGIL 0. HARDON, M.D.
F. W. McRAE, M.D., M’n’g’ng Ed,
JAMES RHARRISON&C]
SUBSCRIPTION t 12.50 A YEAR.
				

